# Targeted *in vitro* suppression of Parkinson’s disease-associated *Desulfovibrio* by selective probiotic and potential probiotic strains

**DOI:** 10.3389/fmicb.2026.1925465

**Published:** 2026-09-21

**Authors:** Sima Talebzadeh, Per Erik Joakim Saris

**Affiliations:** Faculty of Agriculture and Forestry, Department of Microbiology, University of Helsinki, Helsinki, Finland

**Keywords:** antimicrobial activity, *Desulfovibrio*, gut microbiota, Parkinson’s disease, probiotics

## Abstract

**Inroduction:**

Emerging evidence links fecal *Desulfovibrio* (DSV) abundance with Parkinson’s disease (PD) severity. While probiotics may relieve symptoms, their direct effects on DSV remain unexplored.

**Methods:**

This study evaluated the inhibitory potential of 32 probiotic and potential probiotic strains against three DSV strains isolated from three individual PD patients. Antimicrobial activity was quantified using agar well diffusion and microplate assays, and the operational minimum inhibitory con centration (MIC₉₀) was determined based on the total crude dry matter content of cell-free supernatants (CFS).

**Results:**

All tested lactic acid bacteria (LAB) strains, except *Limosilactobacillus reuteri* Hambi410, inhibited DSV. Quantitative micro assay results showed that *Lacticaseibacillus rhamnosus* LGG exhibited the highest inhibition (up to 44.82% ± 8.31%) after neutralization and catalase treatment. Among the eight tested *Bifidobacterium* strains, *Bifidobacterium bifidum* CUL-17 and *Bifidobacterium longum* E-96664 exhibited inhibitory effects comparable to those observed in LAB. The MIC₉₀ values revealed that *Lactobacillus acidophilus* Hambi1448 and *Lactiplantibacillus plantarum* E98 were the most potent, with MIC₉₀ values ranging from 25.2 to 29.8 mg/mL. Spearman’s correlation confirmed a significant inverse relationship between initial pH and inhibition zones (*p* < 0.001). Crucially, analysis of covariance (ANCOVA) demonstrated that strain specific inhibitory variations remained highly significant (*p* < 0.005) after statistically controlling for the confounding effect of acidification, which is consistent with the involvement of non-acidic bioactive metabolites. Probiotic Bacillus strains showed no direct inhibition but are discussed for their role in alleviating PD-related constipation.

**Discussion:**

These findings demonstrate that specific probiotics can target PD-derived DSV, offering a novel strategy for microbiome-based PD management.

## 1 Introduction

Parkinson’s disease (PD) is a prevalent neurodegenerative disorder characterized by the progressive loss of dopaminergic neurons and the intracellular accumulation of toxic fibrils of neuronal protein α-synuclein (α-syn) (Surmeier et al., 2017). Moreover, gastrointestinal symptoms, dysfunction and alterations in gut microbiota often preceded motor symptoms in PD patients, even by a decade or two, emphasizing the importance of early gut-based interventions (Scheperjans et al., 2018). Recent studies, including Murros et al. (2021) have highlighted the association of gut microbiota, particularly *Desulfovibrio* (DSV) bacteria, with Parkinson’s disease, underscoring the growing interest in the role of gut microbes in neurodegenerative disorders. According to Braak’s hypothesis (Braak et al., 2003), PD may originate outside the central nervous system, potentially in the gastrointestinal tract, where a pathogen/toxin crosses the mucosal barrier and invades the enteric nervous system and may reach vulnerable brain regions via the vagus nerve, initiating the characteristic progressive neurodegeneration observed in the disease.

The sign of intestinal inflammation is observed frequently in individuals with PD, which is often associated with a notable shift in gut microbial composition and characterized by an increased abundance of pro-inflammatory, lipopolysaccharide-producing bacteria and reduced level of beneficial microbes (Leta et al., 2021). Such alterations may compromise the gut barrier and contribute to the accumulation of α-syn, allowing bacterial products and metabolites to influence brain function through the gut–brain axis. This axis functions as a bidirectional communication system that integrates neural, hormonal, and immune pathways, thereby linking gut health directly to neurodegeneration. Similarly, research in our own group has identified a significantly higher abundance of DSV species, in the gut microbiota of PD patients compared to healthy individuals (Murros et al., 2021). Additionally, the severity of symptoms correlated with the abundance of DSV in the PD patients. When DSV isolated from PD patients and healthy individuals were fed to PD-model *Caenorhabditis elegans* nematodes, only the DSV strains from PD patients caused significant α-syn aggregation (Huynh et al., 2023). DSV are sulfate-reducing bacteria known for producing hydrogen sulfide (H₂S), a metabolite that, in excessive amounts, can disrupt mitochondrial function, induce inflammation, and impair both intestinal and neuronal integrity, thereby possibly contributing to neurodegeneration in individuals (Murros et al., 2021). Hydrogen sulfide can also paralyze the intestinal muscles leading to constipation, which about 80% of PD patients suffer from (Munteanu et al., 2024). These findings suggest that selectively targeting DSV could provide a promising therapeutic strategy for reducing gut-originated inflammation and its downstream neurological consequences.

Probiotics, defined as live microorganisms that confer health benefits to the host when administered in adequate amounts (WHO, 2002), are increasingly recognized for their potential to restore microbial balance in PD-associated dysbiosis. Clinical studies have shown beneficial effects of probiotic supplementation in PD patients through a 12-week randomized, double-blind, placebo-controlled trial, including improvements in gastrointestinal and motor symptoms, oxidative stress, and reducing inflammation markers (Tamtaji et al., 2019). Researchers demonstrated that a multi-strain probiotic formulation, including *Lactobacillus* and *Bifidobacterium* species can reduce intestinal and neuroinflammation, enhance neurotrophic factors, and improve both motor and non-motor symptoms in a PD mouse model (Leta et al., 2021).

A notable example of a dual-function probiotic is *Limosilactobacillus fermentum* ME-3, which has been shown to possess both strong antimicrobial properties against intestinal pathogens and high antioxidant activity. This unique strain, developed and patented in Estonia, demonstrated the ability to reduce *Salmonella* infection and associated inflammation in animal models. In human clinical trials, supplementation with *L. fermentum* ME-3 improved serum antioxidant status, optimized lipid metabolism by enhancing LDL composition, and reduced oxidative stress, highlighting its potential as an anti-atherogenic and health-promoting (Mikelsaar and Zilmer, 2009). These results further support the potential of probiotics as an adjunctive strategy for modulating inflammation and gut microbiota in PD, but, specifically, no study to date has evaluated probiotic candidates for their targeted capacity to inhibit DSV strains derived directly from Parkinson’s disease patients.

Specific probiotic formulations have demonstrated neuroprotective effects in Parkinson’s disease models. For example, the commercial multi-strain probiotic formulation named SLAB51, consisting of eight bacterial strains, has been shown to reduce inflammation through modulation of cytokine production and attenuation of oxidative stress, thereby contributing to improved intestinal and neuronal homeostasis (Bonfili et al., 2018). Furthermore, SLAB51 has been identified as a promising candidate for preventing or complementing treatment for PD (Castelli et al., 2020). Notably, in PD models, SLAB51 has shown the ability to modulate gut-brain axis signaling, reduce α-syn aggregation, and restore dopaminergic neuronal function. These effects enhance antioxidant defenses and exert neuroprotective effects, indicating its therapeutic potential in neurodegenerative conditions such as PD and AD. Together, these findings suggest that probiotics may offer a supportive therapeutic strategy for modulating the gut–brain axis in neurodegenerative disorders.

To address this knowledge gap, the present study evaluated the direct *in-vitro* inhibitory effects of a comprehensive library of 32 probiotic and potential probiotic strains against three distinct patient-derived DSV isolates from three individual PD patients. By uncoupling organic acid-driven acidification from independent metabolic inhibition, we characterize specific high-potency candidates that offer a novel, targeted microbiome-based strategy for the therapeutic management of PD.

## 2 Materials and methods

### 2.1 Bacterial strains and cultivation conditions

Three clinical *Desulfovibrio* species *D. desulfuricans* (DES 1), *D. fairfieldensis* (DES 4), and *D. piger* (DES 10) were utilized in this study. These strains were previously isolated from fecal samples of three individual PD patients, characterized biochemically and genetically, and maintained in the microbial collection of Prof. Per Saris (Department of Microbiology, University of Helsinki) (Huynh et al., 2023; Murros et al., 2021). These specific isolates were selected as target strains because they represent the key H_2_S producing DSV species implicated in PD-associated gut dysbiosis and have been functionally shown to induce α-syn aggregation in animal models (Huynh et al., 2023). The liquid Postgate medium (DSMZ medium 63) used for bacterial inoculation took place within an anaerobic workstation (Don Whitley Scientific, West Yorkshire, UK). The bacterial cultures were maintained under anaerobic conditions at 37 °C. Inside the anaerobic jar, anaerobic conditions were sustained utilizing the Microbiology Anaerocult® A system (Merck KGaA, Darmstadt, Germany), as confirmed by an Anaerotest® strip (Merck KgaA). Bacterial growth was monitored by the formation of ferrous sulfide (black precipitate) within three to 7 days.

A total of 32 strains were tested that are listed in Table 1 and provided by Prof. Per Saris bacterial collection (Department of Microbiology, University of Helsinki) and propagated anaerobically in 5 mL De Man, Rogosa, and Sharpe (MRS) broth (NEOGEN culture media, United States) and Difco Luria Bertani broth Lennox (Difco, United States) for *Bacillus* bacteria at 37 °C for 24–48 h.

**Table 1:** List of tested probiotic bacterial strains along with their origin.

No.	Bacterial strains	Origin
1	*Lacticaseibacillus rhamnosus* ATCC53103 (LGG)	Gefilus drink
2	*Lacticaseibacillus rhamnosus* 705	Valio Ltd.
3	*Lacticaseibacillus casei* DN-114001	Isolate from Actimel yoghurt (Danone)
4	*Lactobacillus acidophilus* La5	TeknoFood/Danisco
5	*Lactobacillus acidophilus* Hambi1448	Robert Harper <-145Visby
6	*Lactobacillus acidophilus* 74–2	TeknoFood/Danisco
7	*Lactiplantibacillus plantarum* HC1775	Creative enzyme
8	*Lactiplantibacillus plantarum*	VTT culture collection number E-981138
9	*Lactiplantibacillus plantarum* E76	VTT culture collection number E-78076
10	*Lactiplantibacillus plantarum* SM34	Guesh Mulaw from Ethiopia
11	*Ligilactobacillus salivarius* LS86	Shanghai Novanat Co., Ltd
12	*Ligilactobacillus salivarius* ATCC11742	Hansen Co.
13	*Limosilactobacillus fermentum* ATCC14931	Hansen Co.
14	*Limosilactobacillus reuteri* PL111	Nazar Hussain (Lab from Pakistan)
15	*Limosilactobacillus reuteri* Hambi410	Chr. Hansen
16	*Lacticaseibacillus paracasei* PL5	Nazar Hussain
17	*Levilactobacillus brevis* L62	Lallemand, Baking Solution Boulangerie
18	*Lactobacillus delbrueckii* PL13	Nazar Hussain
19	*Lactobacillus delbrueckii* PL11	Nazar Hussain
20	*Lacticaseibacillus johnsonii* PL105	Nazar Hussain
21	*Bifidobacterium bifidum* CUL-17	Replete
22	*Bifidobacterium bifidum* SAA349	Duo+ probiotic product
23	*Bifidobacterium longum subsp. longum*	VTT Strain number E-96664 T
24	*Bifidobacterium longum* 420	TeknoFood
25	*Bifidobacterium longum subsp. infantis*	VTT Strain number E-97796 T
26	*Bifidobacterium longum subsp. infantis* SAA352	DDS Junior probiotic product
27	*Bifidobacterium infantis/lactis* SAA347	Probiottibaby product, Bioteekki company
28	*Bifidobacterium adolescentis* DSM20083	Added by J. Arpidinen
29	*Bacillus subtilis* HU58	www.dralexrinehart.com
30	*Bacillus subtilis* CU1	www.supersmart.com
31	*Bacillus coagulans* SAA937	Probiotic capsules named Gastronel
32	*Bacillus coagulans* MTCC5856	www.afisupplements.com

All bacterial strains used in this study are available upon reasonable request for non-commercial research purposes, subject to applicable national and international regulations.

### 2.2 Preparation of standardized *Desulfovibrio* cell suspensions

To ensure a standardized inoculum for all antimicrobial assays, firstly DSV cultures were harvested, washed, and resuspended following the protocol described by Bernardez and de Andrade Lima (2015), with minor modifications. Briefly, 4.5 mL of DSV culture were mixed with 0.2 mL hydrochloric acid and incubated for 15 min before centrifugation at 10 °C for 20 min at 1560 g. The pellet was washed twice with distilled water (10 mL each), including a second centrifugation step at 12860 g for 10 min. The final biomass was resuspended in 10 mL of distilled water to prepare a start bacterial suspension (Bernardez and de Andrade Lima, 2015). Bacterial suspensions were subsequently adjusted to the desired optical density (OD_600_) of 0.1 to obtain a standardized inoculum before use in the antimicrobial assays. Further dilutions (1:10) were made if needed for use in the assays and this dilution factor was considered in the measurement of the DSV concentration.

### 2.3 pH measurement and CFS preparation

The pH of all CFSs was determined using a pH-meter (Thermo Orion, 420A+). Calibration of the pH-meter was performed using buffer solutions with pH values of 4, 7, and 10. Subsequently, active cultures divided in two parts, one part was utilized to determine the final bacterial biomass density at OD_600_ using a Biophotometer (Eppendorf AG 22331, Hamburg), confirming that the cell concentrations were standardized to approximately 10^7^ CFU/mL for subsequent analysis. The remaining portion was subjected to centrifugation at 10000 g for 15 min at 4 °C to harvest the cell-free supernatant (CFS) and it was also filtered through a 0.22 μm BIOFIL syringe filter yielding CFSs. To find suitable bacteria that are able to produce inhibitory compounds (in addition to producing acid and hydrogen peroxide) against DSV bacteria, in the first step, all CFSs were neutralized by adding NaOH and catalase enzyme as follows. The pH of the CFSs was adjusted to 7.0 using a 4 M NaOH and pH meter. Then, to remove hydrogen peroxide, neutralized CFSs were treated with 0.1 mg/mL bovine catalase (Sigma-Aldrich Corporation, United States) at 25 °C for 30 min and then filter-sterilized. The CFSs were either used immediately for antimicrobial testing or frozen −70 °C before usage.

### 2.4 Investigating the inhibitory activity of treated CFS’S of probiotics against the DSV bacteria

#### 2.4.1 Agar well diffusion method

Antimicrobial activity of lactobacilli strains against DSV was determined using the agar well diffusion assay. Postgate agar plates (80 mm diameter) were used for this assay. DSV strains with $10^{7}$ CFU/ml concentration were spread onto the surface of the Postgate agar plate. The wells (6 mm) were made in the agar layer, and 100 μL of each sample supernatant were applied per well and allowed to diffuse at 37 °C for 1 h. Plates were incubated at 37 °C for 72 h and antimicrobial activity was determined by measuring the clear inhibition zone around each well. Sterile MRS broth and Metronidazole (16 μg/mL) (Thermo Fisher, US) were used as negative and positive controls, respectively. All antimicrobial assays were performed using three independent biological replicates, each prepared with triplicates as technical replicates (yielding n = 9 total observations per strain) to ensure statistical reliability of the ANOVA and ANCOVA error terms. The diameter of the inhibition zones was measured in millimeters using a standard ruler with an accuracy of 0.1 mm.

#### 2.4.2 Microplate assay

In the second step, antimicrobial activity of the treated CFSs was determined using microplate assay (Lim, 2016). For this purpose, 100 μL of treated CFSs, and 100 μL adjusted suspension (1.0 × $10^{6}$ CFU/ml) of the DSV bacteria were poured on each well. A negative control containing MRS broth and PG, a positive control containing MRS broth and each DSV bacteria, and a CFS control containing each CFS and PG broth were used. After that, the microplate was incubated at 37 °C for 48 h under anaerobic conditions using airtight chambers with Anaerocult A gas packs. The optical density (OD) at 600 nm was measured using a microplate reader (Infinite M200, Tecan, USA). Each experiment was performed using three independent biological replicates, with each condition tested in technical triplicates. For the evaluation of the inhibition of treated CFS, the percentage of growth at each strain’s CFS was calculated using the following equation (Jalaei et al., 2014):

$$\%\text{Inhibition}=100-[\frac{\begin{array}{l} \mathrm{OD}\;\text{of sample}-\mathrm{OD}\;\text{of}\\ \text{background control} \end{array}}{\begin{array}{l} \mathrm{OD}\;\text{of growth control}-\\ \mathrm{OD}\;\text{of background control} \end{array}}\times 100]$$

Finally, 14 strains with highest inhibitory effect on three DSV bacteria were selected as protective cultures and subjected to further investigation.

### 2.5 Determination of dry matter concentration of CFS

The concentration of cell-free supernatant (CFS) was determined based on its dry matter content using a gravimetric drying method (Lambert and Pearson, 2000). Briefly, a known volume of CFS (2 mL) was transferred into a pre-weighed petri dish that had been previously dried at 105 °C and cooled in a desiccator. The dish containing the sample was weighed and then dried in a hot-air oven (Memmert U25, Germany) at 105 °C until constant weight. After drying, the dish was cooled in a desiccator and weighed again to determine the dry residue.

The dry matter of CFS was calculated using the following equation:

$$\mathrm{CFS}\;\text{concentration}\;(\mathrm{mg}/\mathrm{mL})=\frac{\mathrm{dry}\;\text{weight of residue}\;(\mathrm{mg})\;}{\text{volume of}\;\mathrm{CFS}\;\text{sample}\;(\mathrm{mL})}$$

Where the weight of dried residue corresponds to the difference between the weight of the dish containing the dried sample and the weight of the empty dish. The obtained value was used to express the operational concentration of crude CFS in mg/mL, for subsequent serial dilution in the MIC₉₀ assay. It is acknowledged that this value reflects total crude dry residue including residual medium constituents and purified active compounds.

### 2.6 Minimum inhibitory concentration of CFS’s

The MIC₉₀ of CFS for protective bacteria was determined using the conventional broth microdilution technique. Briefly, 100 μL of PG broth was poured into each well, and then 100 μL of the CFS were poured in the first well. After pipetting, 100 μL of suspension were transferred from the first well to the second well. It was thus diluted to 5 wells in this manner. The final volume of each well reached 100 μL. Finally, 10 μL of the DSV bacterial suspension (5.0 × $10^{6}$ CFU/ml) were added to each well. A negative control containing MRS broth and PG broth, a positive control containing MRS broth and each DSV bacteria, and a CFS control containing each CFS and PG broth were also included. The microplates were incubated in anaerobic conditions at 37 °C for 48 h. The percent of inhibitory effect of each concentration of CFSs was determined as described above. The MIC₉₀ was defined as the lowest CFS dry matter concentration (mg/mL) that resulted in ≥90% growth inhibition compared to the positive control. For strains that did not reach 90% inhibition within the tested concentration range, the MIC₉₀ was reported as greater than the highest tested concentration (> 40.0 mg/mL) rather than estimated by extrapolation. Each assay was carried out across three independent biological replicates, with internal technical triplicates, and for validation of the experimental procedures, sterility controls of culture media, growth controls of DSVs, and positive inhibition controls using metronidazole (16 μg/mL) (Thermo Fisher, US) were included in all assays.

### 2.7 Statistical analysis

All experiments were conducted using three independent biological replicates, with each assay performed in technical triplicates. Data are presented as mean ± standard deviation (SD) of the independent biological runs. Statistical significance was evaluated using GraphPad Prism (version 9.0) and SPSS (version 28.0). To evaluate the targeted inhibitory effects of cell-free supernatants (CFS) while rigorously uncoupling the confounding effect of organic acid-driven acidification, Analysis of Covariance (ANCOVA) was performed, wherein the pH of the CFS was designated as the covariate. Prior to ANCOVA execution, the critical assumptions of normality (Shapiro–Wilk test), homogeneity of variances (Levene’s test), and homogeneity of regression slopes were tested and successfully satisfied (*p* > 0.05). Post-hoc comparisons of the adjusted means were determined using Tukey’s Honestly Significant Difference (Tukey’s HSD) test. *p*-values of less than 0.05 (*p* < 0.05) were considered statistically significant.

## 3 Results

### 3.1 Growth characterization and acidification profiles of bacterial strains

To elucidate the dynamics between bacterial growth and organic acid production across acidifying taxonomic groups, a regression analysis was performed on the LAB and *Bifidobacterium* strains ( n=28 ) (Figure 1). Excluding the phylogenetically distinct *Bacillus* isolates (which maintained near-neutral pH values between 6.31 and 7.74), the constructed model demonstrated a strong negative and statistically significant negative linear relationship between bacterial biomass density and final pH ( $R^{2}=0.388$ , F(1,26)=16.44 , P<0.001 , β=−0.622 ), confirming that higher growth density independently contributes to increased medium acidification within these lactic/acetic acid-producing fermenters. *Bacillus* strains were evaluated separately due to their distinct, non-acidifying physiological behavior.

**Figure 1:**
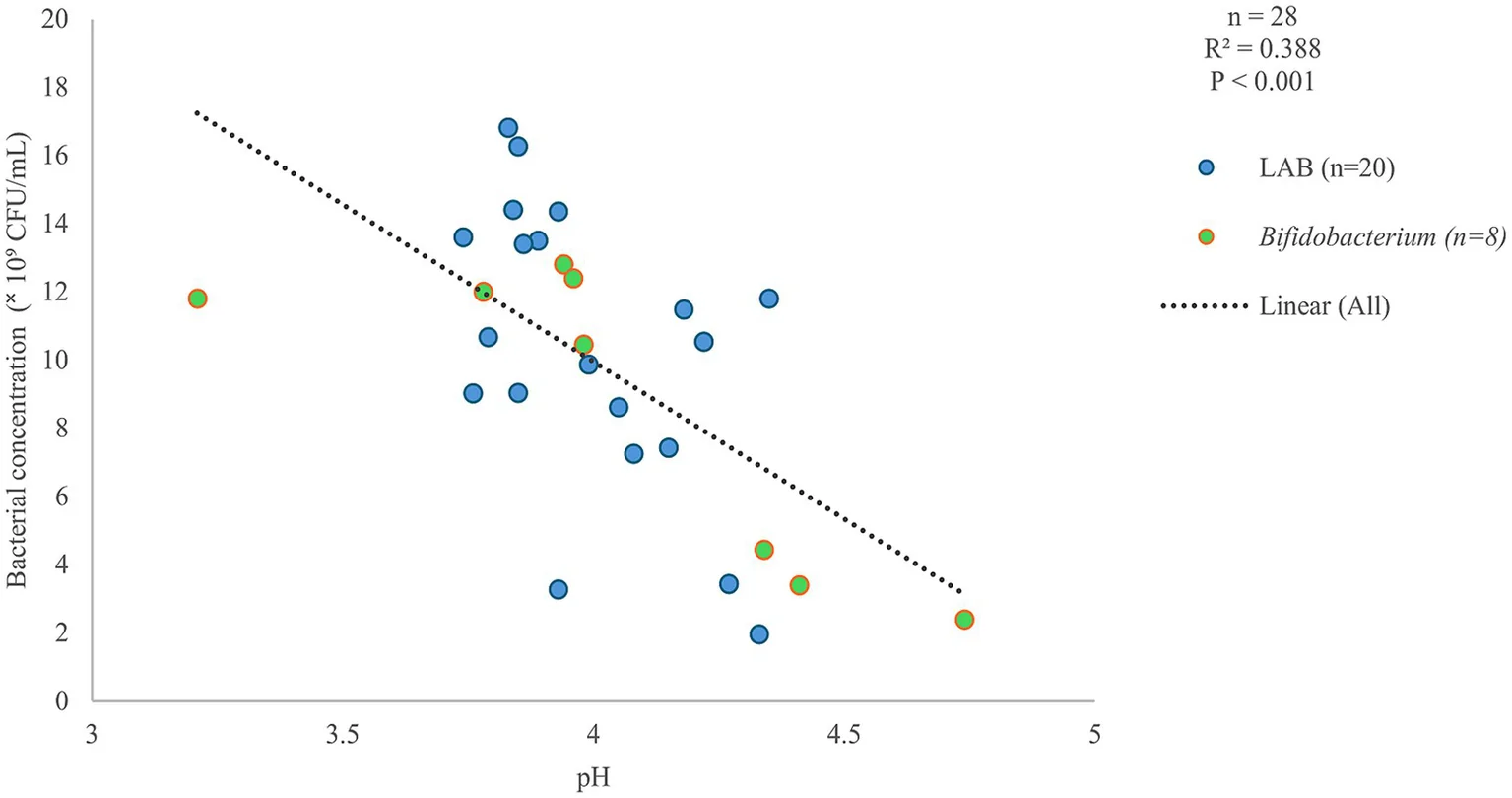
Relation between bacterial concentration and pH.

### 3.2 Inhibition of *Desulfovibrio* by cell-free supernatant of probiotic strains

#### 3.2.1 Screening of antimicrobial activity of CFS via agar well diffusion assay

The inhibitory spectrum of the 32 probiotic and potential probiotic CFS against three isolated DSV strains (DES1, DES4, and DES10) was quantified by measuring the inhibition zone diameters across nine independent replicates. A two-way analysis of variance (ANOVA) revealed highly significant main effects for both the probiotic strains [*F*(31, 768) = 1412.35, *p* < 0.001] and the target pathogenic strains [*F*(2, 768) = 84.12, *p* < 0.001]. Furthermore, a significant probiotic, pathogen interaction effect was established [*F*(62, 768) = 14.56, *p* < 0.001], indicating that the antimicrobial efficacy of specific CFS is highly dependent on the target DSV strain. To account for variations in initial biomass accumulation among the 32 strains and its potential confounding effect on zone diameters, an analysis of covariance (ANCOVA) was executed, utilizing the mean bacterial CFU as a covariate. The ANCOVA model demonstrated that even after statistically adjusting for and neutralizing the effect of biomass density, the inherent inter-strain differences in antimicrobial efficacy remained highly significant [*F*(31, 767) = 284.14, *p* < 0.001]. This indicates that the superior inhibitory capacity of leading strains is driven by the intrinsic potency and quality of their bioactive metabolites rather than a simple consequence of higher cell density. Tukey’s HSD post-hoc comparisons demonstrated distinct hierarchical profiles of inhibition across taxonomic groups. LAB strains exhibited the most potent antimicrobial activities. Among them*, Lc. rhamnosus* GG (LGG) elicited the most pronounced antagonistic activity, yielding maximum inhibition zones of 6.37 ± 0.4 mm against DES 10, followed by 5.89 ± 0.3 mm against DES 1, and 5.78 ± 0.3 mm against DES 4, significantly outperforming 85% of the tested library (*p* < 0.05). Other notable inhibitors included *L. acidophilus* Hambi1448 and *Lp. plantarum* E98, which maintained consistently high zones of inhibition across all three pathogens (ranging from 4.1 to 5.7 mm) with no statistically significant variance between their respective inter-pathogen yields.

Within the *Bifidobacterium* genus, a heterogeneous inhibitory trend was observed. *B. bifidum* CUL-17 and *B. longum* E-96664 exhibited substantial, statistically equivalent antagonistic activity to the leading LAB strains (mean zones ˃ 4.2 mm, *p* > 0.05). In contrast, other *Bifidobacteria*, such as *B. infantis/lactis* SAA347, demonstrated significantly lower efficacy (mean zones ≤ 1.5 mm, *p* < 0.01). Crucially, all four tested *Bacillus* strains (*B. subtilis* CU1, *B. subtilis* HU58, *B. coagulans* MTCC5856, and *B. coagulans* SAA937) completely failed to produce any visual zones of inhibition (0.00 mm) across all nine replicates, establishing a highly significant metabolic divergence (*p* < 0.001) from the acid-producing groups regarding direct competitive exclusion in solid media Figure 2.

**Figure 2:**
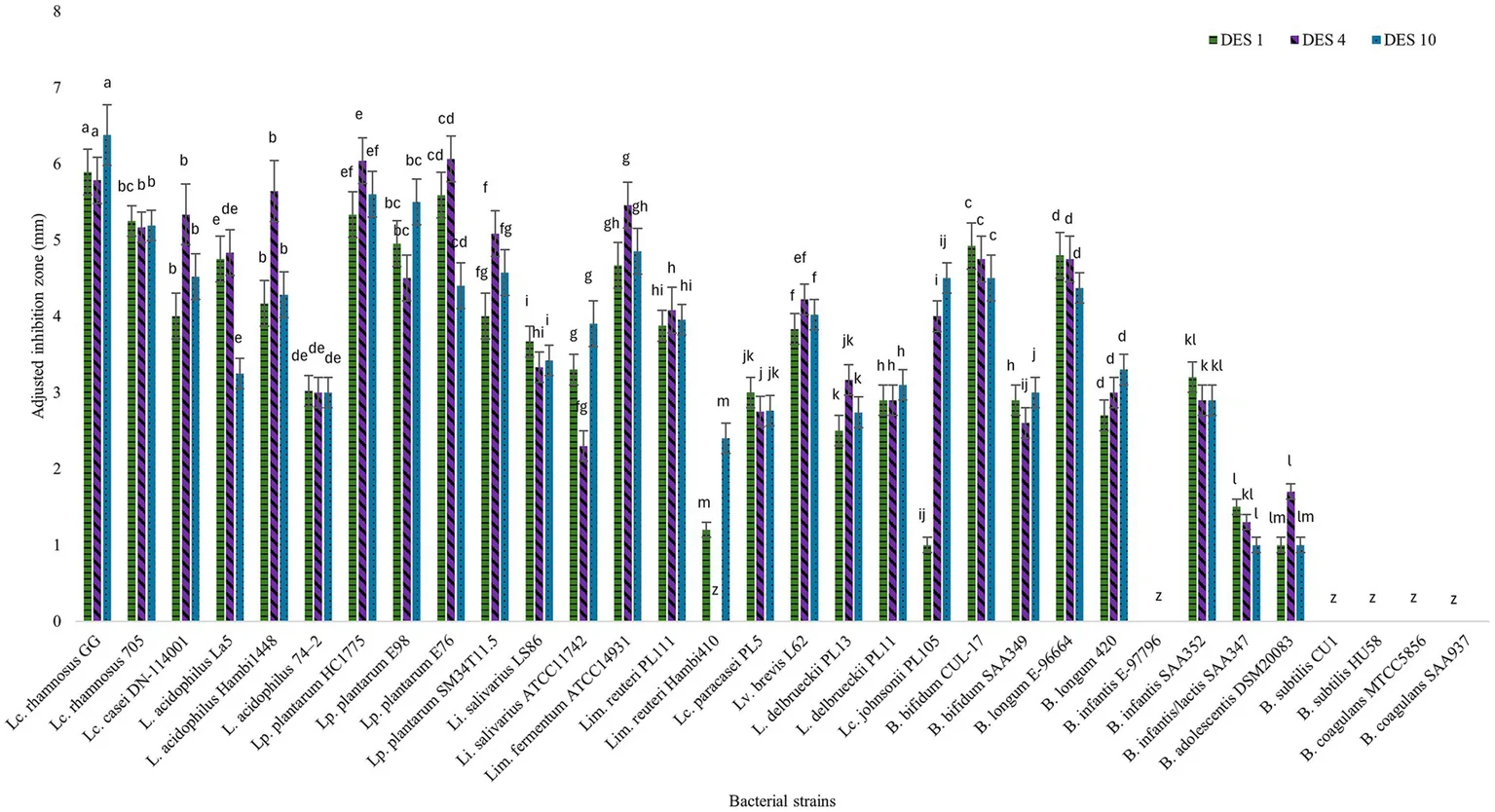
Adjusted anti-DSV efficacy of probiotic CFS quantified via agar well diffusion assay. Data are presented as means ± standard deviation (SD) from three independent biological experiments, each performed in technical triplicates (*n* = 9 total replicates) after statistically neutralizing the confounding effect of initial bacterial biomass (CFU/mL) via analysis of covariance (ANCOVA). Different letters above the bars indicate statistically significant differences between probiotic strains within the same target pathogen cluster according to Tukey’s HSD post-hoc test (*p* < 0.05).

#### 3.2.2 Quantitative assessment of anti-DSV efficacy via microplate assay (neutralized and catalase-treated CFS)

The inhibitory effects of CFS of 14 selected probiotic strains, following the removal of organic acids and hydrogen peroxide, are presented in Figure 3. To elucidate the contribution of non-acidic antimicrobial compounds (e.g., bacteriocins and bioactive peptides) secreted by the top-performing probiotic strains, a quantitative microplate inhibition assay was conducted using CFS subjected to pH neutralization and catalase treatment. A two-way ANOVA conducted on the neutralized data revealed highly significant main effects for the probiotic strains [*F*(13, 84) = 112.45, *p* < 0.001] and the target pathogenic DSV strains [*F*(2, 84) = 8.92, *p* = 0.002]. Furthermore, a significant probiotic, pathogen interaction effect was established [*F*(26, 84) = 3.14, *p* = 0.008], confirming that the non-acid-dependent antagonistic yield is highly specific to the target pathogen strain. Post-hoc comparisons via Tukey’s HSD test demonstrated that, *Lc. rhamnosus* GG (LGG) maintained the most robust non-acidic inhibitory profile across the board. LGG achieved its peak quantitative efficacy against the pathogenic strain DES 10, resulting in an inhibition rate of 44.82% ± 8.31%, followed by 41.36% ± 7.97% against DES 1 and 39.55% ± 8.56% against DES 4. This anti-DSV capacity was statistically equivalent to the leading *Bifidobacterium* strains, notably *B. bifidum* CUL-17 (which maintained a high inhibition range of 40.5 to 45.1% across the pathogens). Conversely, for the majority of the remaining tested strains, the removal of organic acids and hydrogen peroxide led to a substantial reduction in inhibitory capacity, with mean inhibition percentages dropping below 25%, establishing that while acidic dynamics dominate the screening phase, leading strains like LGG possess distinct, intrinsically potent non-acidic bioactive principles.

**Figure 3:**
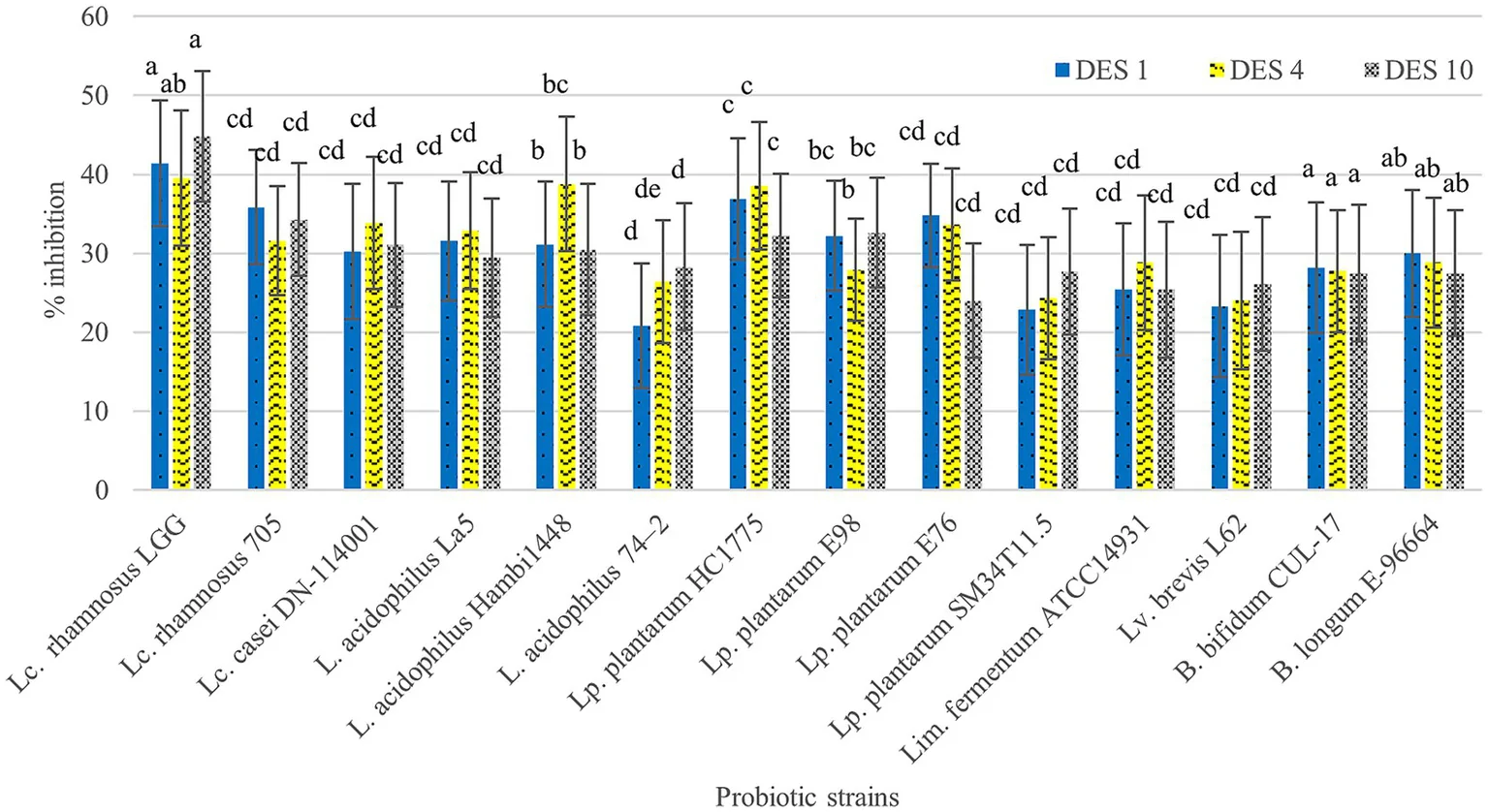
Percentage of inhibition of cell free supernatant of selected bacteria against three DSV bacteria. Different letters (a–d) above the bars indicate statistically significant differences between probiotic strains within the same target pathogen cluster according to Tukey’s HSD post-hoc test (*p* < 0.05).

### 3.3 Determination of MIC₉₀ of CFS of protective bacteria against three DSV bacteria

MIC₉₀ values were determined using a broth microdilution assay with serial dilutions of CFS. Growth inhibition was measured across the tested concentration range. For strains that did not achieve 90% inhibition within the tested concentration range, MIC₉₀ values were designated as exceeding the maximum tested concentration (> 40.0 mg/mL) rather than extrapolated. The results of the MIC₉₀ of each CFS of protective bacteria are shown in Table 2. A comparative analysis of MIC₉₀ values revealed notable variation in the inhibitory activity of the tested probiotic strains against *Desulfovibrio* isolates. Among the strains, *L. acidophilus* Hambi1448 and *Lp. Plantarum* E98 and, *Lc. rhamnosus* GG exhibited the strongest inhibitory effects, with the lowest MIC₉₀ values ranging from 25.2 to 29.8 mg/mL across all tested isolates. In contrast, *B. bifidum* CUL-17 and *B. longum* E-96664 showed comparatively weaker activity, with MIC₉₀ values exceeding 38 mg/mL. Most *Limosilactobacillus reuteri* and *Lc. casei* strains demonstrated moderate inhibitory effects, with MIC₉₀ values generally between 30 and 37 mg/mL. Overall, the results indicate strain-dependent variability in antimicrobial activity, highlighting specific candidates with enhanced potential for inhibiting *Desulfovibrio* growth.

**Table 2:** MIC_90_ (mg/mL) of crude CFS dry matter of the protective bacteria against three DSV bacteria (mean, *n* = 3).

Probiotic bacterial strains	MIC₉₀ (mg/mL)		
	DES 1	DES 4	DES 10
*Lacticaseibacillus rhamnosus* LGG	29.8	28.6	29.0
*Lacticaseibacillus rhamnosus* 705	34.7	36.1	35.4
*Lacticaseibacillus casei* DN-114001	37.8	37.4	35.5
*Lactobacillus acidophilus* La5	35.1	34.9	35.3
*Lactobacillus acidophilus* Hambi1448	25.5	27.4	29.5
*Lactobacillus acidophilus* 74–2	37.5	35.2	> 40.0
*Lactiplantibacillus plantarum* HC1775	37.9	36.1	> 40.0
*Lactiplantibacillus plantarum* E98	25.2	28.6	27.6
*Lactiplantibacillus plantarum* E76	32.2	32.9	34.8
*Lactiplantibacillus plantarum* SM34	38.2	> 40.0	36.2
*Limosilactobacillus fermentum* ATCC14931	38.5	37.8	37.9
*Levilactobacillus brevis* L62	36.2	37.2	35.2
*Bifidobacterium bifidum* CUL-17	> 40.0	38.9	39.5
*Bifidobacterium longum* E-96664	39.0	> 40.0	39.3

### 3.4 Effect of pH of CFS on DSV inhibition

The effect of pH on the antimicrobial characteristics of probiotic CFS against DSV was assessed. All samples before and after adjustment to pH 7 were evaluated for their residual anti-DSV activities (Figure 4). The antagonistic effects were drastically diminished upon neutralization with alkali, demonstrating that environmental acidification and organic acid production represent a primary mechanism of DSV suppression. However, inhibitory activity was not completely abolished in several potent strains (e.g., *Lc. rhamnosus* GG and 705), indicating a dual mode of action where persistent residual inhibition operates alongside organic acid-driven acidification. To evaluate the relationship between the initial pH of the CFS and the resulting inhibition zones, a Spearman’s rank correlation analysis was performed across all experimental replicates. In contrast to the initial tentative analysis, a highly significant, moderate negative correlation was unveiled (*p* = −0.485, *p* < 0.001). This statistical significance confirms that lower pH values (higher acidity) are fundamentally associated with enhanced descriptive zones of inhibition. To further isolate the specific inhibitory potential of the strains independent of their acid production, an analysis of covariance (ANCOVA) was executed, utilizing the initial pH as a covariate. The ANCOVA model revealed that even after statistically controlling for the confounding effect of pH (*p* < 0.001), the therapeutic variability among the probiotic strains remained highly significant (*p* < 0.005). This critical statistical validation indicate that the superior antimicrobial efficacy of specific strains is not merely an artifact of organic acid accumulation, supporting the presence of distinct, strain-specific bioactive components.

**Figure 4:**
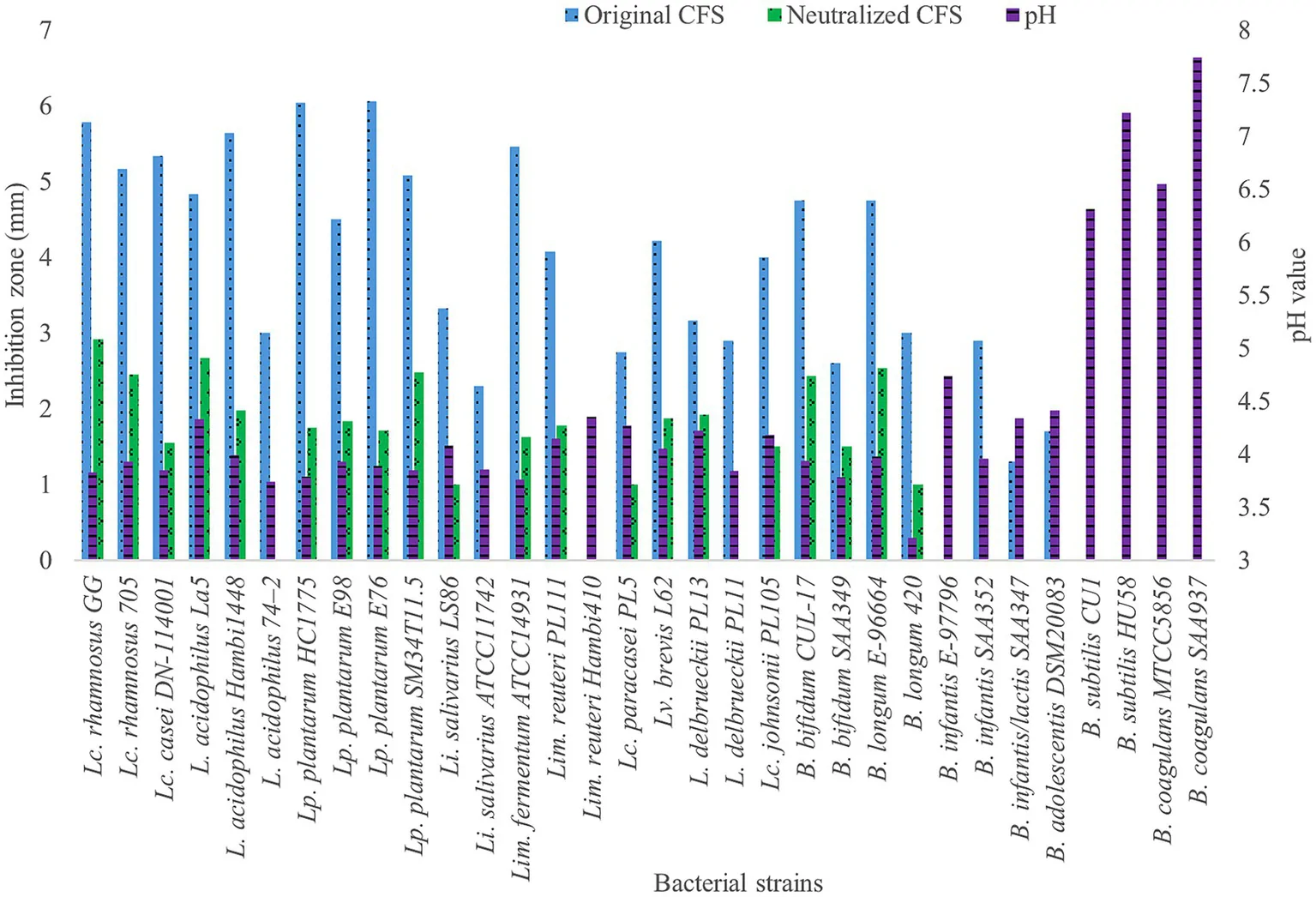
Dual-axis representation of probiotic CFS anti-DSV activity in relation to initial pH and neutralization states. Bars indicate the mean inhibition zone diameters (mm) of original CFS (purple) and neutralized CFS (blue) corresponding to the left y-axis. Green bars depict the mean initial pH values of the respective strains corresponding to the right y-axis.

## 4 Discussion

In the present study, we evaluated the targeted *in vitro* suppression of PD-associated DSV strains (*D. desulfuricans* DES 1, *D. fairfieldensis* DES 4, and *D. piger* DES 10) by a broad panel of 32 probiotic and potential probiotic strains. Emerging evidence strongly links the fecal abundance of DSV species with PD and the clinical severity of the disease (Murros et al., 2021). DSV species are thought to contribute to neurodegenerative pathology primarily through the generation of excessive hydrogen sulfide (H₂S) and lipopolysaccharides (LPS), which together compromise intestinal epithelial barrier integrity, promote mucosal and systemic inflammation, and trigger the pathologic aggregation of α-syn within the enteric nervous system (Huynh et al., 2023; Munteanu et al., 2024). While general probiotic supplementation has shown clinical promise in managing non-motor symptoms of PD (Barichella et al., 2016; Leta et al., 2021; Tamtaji et al., 2019), this study provides the first targeted evaluation of probiotic candidates against patient-derived DSV isolates, offering a potential directed probiotic-based therapeutic strategy.

A central finding of our investigation is that the antimicrobial activity against clinical DSV strains is highly strain-dependent and driven by both organic acid production and independent bioactive metabolites. Primary screening via agar well diffusion demonstrated broad-spectrum activity across lactic acid bacteria (LAB) strains, with *Lacticaseibacillus rhamnosus* LGG generating the largest mean inhibition zones. A key mechanism underlying this initial inhibition is media acidification through the accumulation of organic acids (predominantly lactic and acetic acids) during glucose fermentation. Spearman’s rank correlation analysis confirmed a robust, statistically significant negative correlation between initial CFS pH and inhibition zone diameters (*p* < 0.001), reinforcing the established role of pH reduction in inhibiting sulfate-reducing bacteria.

Overall, these findings demonstrate that organic acid production and concomitant pH reduction serve as the major driver of DSV suppression, while specific high-potency probiotic candidates maintain secondary, non-acidic inhibitory mechanisms at physiological pH. It should be noted as a study limitation that individual commercial organic acids (e.g., pure lactic acid or acetic acid) were not tested in isolation at matching concentrations in the present assays. Consequently, while our statistical models (ANCOVA) evaluate the remaining variance after pH adjustment, direct experimental quantification using purified organic acid controls is necessary in future work to precisely delineate the proportion of inhibition governed solely by specific organic acid molecules versus non-acidic bioactive metabolites.

However, relying solely on acidification as an antimicrobial mechanism is insufficient for gut-targeted therapies, as host buffering capacity in the human colon rapidly neutralizes luminally secreted organic acids. Therefore, identifying strains capable of producing non-acidic bioactive metabolites that remain active at neutral pH is paramount. To rigorously uncouple organic acid-driven acidification from inherent metabolic inhibition, we performed Analysis of Covariance (ANCOVA) using initial pH as a covariate. Crucially, the ANCOVA model demonstrated that even after statistically controlling pH as a covariate (*p* < 0.001), the strain-specific variability in inhibitory efficacy remained highly significant (*p* < 0.005). While ANCOVA alone does not provide biochemical identification of active compounds, this remaining variance is consistent with the hypothesis that non-acidic bioactive metabolites contribute to DSV suppression alongside organic acid-mediated inhibition.

This statistical validation was further supported by our quantitative microplate assay using pH-neutralized and catalase-treated CFS. Upon elimination of organic acids and hydrogen peroxide, top-performing strains retained marked inhibitory yields. Notably, *Lc. rhamnosus* LGG exhibited exceptional non-acidic potency, achieving up to 44.82% ± 8.31% growth inhibition against *D. piger* (DES 10). This strain-specific efficacy was also observed among selected *Bifidobacteria*, including *Bifidobacterium bifidum* CUL-17 and *Bifidobacterium longum* E-96664, which maintained sustained anti-DSV activity post-neutralization. Furthermore, gravimetric MIC₉₀ determination highlighted *Lactobacillus acidophilus* Hambi1448 and *Lactiplantibacillus plantarum* E98 as high-potency candidates, yielding the lowest MIC₉₀ values (25.2 to 29.8 mg/mL dry weight) across all tested DSV isolates. Together, these findings suggest that the superior antagonistic capacity of leading LAB strains may be mediated by intrinsically potent non-acidic compounds such as bacteriocins, microcins, or other low-molecular-weight antimicrobial substances (Diep et al., 2009; Mani-López et al., 2022; Silva et al., 1987).

To contextualize these non-acidic inhibitory dynamics, existing literature on the specific top-performing strains provides valuable mechanical insights. For instance, *Lacticaseibacillus rhamnosus* GG (LGG) is documented to secrete low-molecular-weight, heat-stable bacteriocin-like substances as well as antimicrobial proteins such as p40 and p75, which maintain stability across a relatively broad pH range (Seth et al., 2008; Silva et al., 1987). Furthermore, while catalase treatment in our protocol neutralized hydrogen peroxide ( $H_{2}O_{2}$ ), strains such as *L. acidophilus* Hambi1448 belong to species well-known for $H_{2}O_{2}$ accumulation under microaerophilic conditions, alongside the production of class IIa bacteriocins (e.g., acidocin) whose hydrophobic antibacterial action remains functionally resilient following pH neutralization (Hertzberger et al., 2014). Similarly, *Lactiplantibacillus plantarum* strains (such as E98) frequently harbor inducible plantaricin gene clusters (e.g., *plnEF*, *plnJK*) encoding two-peptide bacteriocins; these peptides retain potent membrane-permeabilizing activity against target strains even at neutral pH, although their binding efficiency can be modulated by electrostatic charge variations induced by environmental pH shifts (Anderssen et al., 1998; Diep et al., 2009). Incorporating these strain-specific literature precedents supports the possibility that the persistent anti-*Desulfovibrio* activity observed in our neutralized CFS assays may be mediated by robust, non-acidic proteinaceous or low-molecular-weight metabolites. However, definitive biochemical proof such as enzymatic degradation via proteinase K digestion, thermal stability testing, and structural fractionation remain necessary in future studies to confirm whether bacteriocins or specific microcin-like peptides are indeed responsible for this non-acidic antagonism.

The susceptibility and resistance profiles observed in our study align with and build upon the sparse existing literature regarding DSV suppression. Sulfate-reducing bacteria, particularly DSV species, are recognized for their intrinsic resistance to various traditional antibiotics and physiological stressors, largely attributed to their robust Gram-negative outer membrane barrier, active efflux pumps, and specialized anaerobic energy metabolic pathways (Gibson et al., 1993; Goldstein et al., 2003). Previous investigations into the microbial inhibition of DSV have primarily highlighted sensitivity to severe acidic environments and short-chain fatty acids (SCFAs), which disrupt transmembrane proton motive forces (Christl et al., 1992). Reports specifically addressing bacteriocin or peptide-mediated antagonism against *Desulfovibrio* remain limited. Nevertheless, LAB are known to produce multiple antimicrobial metabolites, including organic acids, hydrogen peroxide, and bacteriocins, which may act independently or synergistically against susceptible bacteria (Dobson et al., 2012; Plaza-Diaz et al., 2019; Tejero-Sariñena et al., 2012). Our findings extend this literature by showing that while acidification is indeed the dominant inhibitory factor, patient-derived DSV isolates exhibit differential susceptibility to non-acidic cell-free fractions, underscoring that resistance to microbial metabolites varies significantly at the species level.

The observed variation in sensitivity among the three DSV strains highlight strain-dependent susceptibility patterns that are highly relevant to PD pathogenesis. Among the target strains, *D. piger* DES 10 displayed the highest overall sensitivity to the top probiotic CFS candidates (such as *Lc. rhamnosus* GG). Species-level differences in outer membrane lipopolysaccharide (LPS) composition, cell-wall permeability, and enzymatic defense mechanisms against microbial metabolites likely account for these variations. Clinically, different DSV species exhibit distinct genotypic traits and H_2_S production capacities, which directly influence the severity of intestinal mucosal barrier degradation and downstream α -syn aggregation (Huynh et al., 2023; Murros et al., 2021). Therefore, the ability of specific probiotics (e.g., LGG and *L. acidophilus* Hambi1448) to effectively suppress all three clinically isolated species despite their intrinsic physiological differences underlines the potential of these strains as broad-spectrum, targeted candidates for modulating DSV-driven dysbiosis in PD.

The distinct sensitivity profiles observed among *D. desulfuricans* DES 1, *D. fairfieldensis* DES 4, and *D. piger* DES 10 highlight the importance of utilizing multiple patient-derived isolates when evaluating potential therapeutics. Because clinical DSV strains exhibit functional variations in toxic metabolite production and α-syn seeding capacity, the marked susceptibility of *D. piger* DES 10 to neutralized CFS of LGG is particularly significant. Suppressing colonic DSV burden with high-potency non-acidic metabolites could directly attenuate H₂S generation, thereby protecting the mucosal layer and disrupting the early inflammatory cascades implicated in PD progression.

In contrast to LAB and *Bifidobacteria*, none of the four tested *Bacillus* strains (*B. subtilis* CU1, *B. subtilis* HU58, *B. coagulans* MTCC5856, and *B. coagulans* SAA937) displayed direct inhibition zones in agar diffusion assays. This clear physiological divergence indicates that *Bacillus* species do not secrete direct antimicrobial metabolites active against DSV *in vitro*. Nevertheless, spore-forming *Bacillus* probiotics, particularly *Weizmannia coagulans* (*B. coagulans*) remain clinically relevant for supporting gut homeostasis in neurodegenerative condition such as PD (Zhang et al., 2024). Over 80% of PD patients suffer from severe chronic constipation, a symptom linked to H₂S-induced inhibition of colonic motor activity and smooth muscle paralysis (Gallego et al., 2008). *B. coagulans* has been shown to modulate gut microbial composition, enhance intestinal motility, and stimulate the growth of endogenous butyrate-producing taxa (such as *Lachnospiraceae*) (Du et al., 2022; Kang et al., 2021; Sasaki et al., 2020). Short-chain fatty acids (SCFAs), particularly butyrate, play essential roles in reinforcing tight junction proteins, suppressing neuroinflammation, and rescuing nigral dopaminergic neurons from oxidative toxicity (Louis and Flint, 2017; Srivastav et al., 2019). Thus, while *Bacillus* strains do not directly suppress DSV growth, their inclusion in multi-strain probiotic formulations could provide complementary clinical benefits by alleviating GI dysfunction and enhancing gut mucosal repair.

Specific probiotic formulations have demonstrated neuroprotective effects in PD models. For example, the commercial multi-strain probiotic formulation named SLAB51, consisting of eight bacterial strains (including *Streptococcus thermophilus* DSM 32245, *Bifidobacterium lactis* DSM 32246, *Bifidobacterium lactis* DSM 32247, *Lactobacillus acidophilus* DSM 32241, *Lactobacillus helveticus* DSM 32242, *Lactobacillus paracasei* DSM 32243, *Lactobacillus plantarum* DSM 32244, *Lactobacillus brevis* DSM 27961), has been shown to reduce inflammation through modulation of cytokine production and attenuation of oxidative stress, thereby contributing to improved intestinal and neuronal homeostasis (Bonfili et al., 2018). Furthermore, SLAB51 has been identified as a promising candidate for preventing or complementing treatment for PD (Castelli et al., 2020). Notably, in PD models, SLAB51 has shown the ability to modulate gut-brain axis signaling, reduce α-syn aggregation, and restore dopaminergic neuronal function. These effects enhance antioxidant defenses and exert neuroprotective effects, indicating its therapeutic potential in neurodegenerative conditions such as PD and AD. Together, these findings suggest that probiotics may offer a supportive therapeutic strategy for modulating the gut–brain axis in neurodegenerative disorders.

A recognized limitation of the present study is the absence of neutralized pure lactic acid controls at equivalent concentrations during the initial screening phase. Furthermore, we acknowledge that expressing MIC₉₀ values based on crude CFS dry weight (mg/mL) represents a practical and operational metric rather than a direct quantification of pure active antimicrobial metabolites, as crude CFS includes residual inactive nutrients from the culture medium. Although our ANCOVA modeling and persistence of inhibitory activity after neutralization strongly suggested the presence of non-acidic mechanisms, future verification, such as proteinase K sensitivity testing, and HPLC fractionation is required to confirm whether these effects are mediated by proteinaceous bacteriocins or other bioactive metabolites responsible for this anti-DSV activity.

In conclusion, our study demonstrates that targeted inhibition of PD-derived *Desulfovibrio* is achievable using specific probiotic LAB strains. Candidates such as *Lc. rhamnosus* LGG, *L. acidophilus* Hambi1448, and *Lp. plantarum* E98 suppress DSV growth via mechanisms that operate independently of simple acidification. These findings provide a scientific foundation for designing multi-strain probiotic formulations that combine potent DSV-inhibiting LAB with supportive, SCFA-enhancing strains to address both microbial dysbiosis and gastrointestinal pathophysiology in Parkinson’s disease.

## 5 Conclusion

Targeting DSV species is a promising strategy to alleviate Parkinson’s disease symptoms due to their role in gut dysbiosis, inflammation, and neurodegeneration. This study demonstrates that. While organic acid-driven acidification is the primary mode of DSV suppression by probiotic LAB, specific strains exert persistent anti-DSV activity even after pH neutralization, highlighting a complementary mechanism likely involving bioactive metabolites that remain active at physiological pH. Notably, while *Bacillus* strains did not directly inhibit DSV, their documented clinical efficacy in stimulating endogenous butyrate production and relieving constipation makes them valuable components for a multi-strain probiotic complex. We propose that a formulation combining potent LAB inhibitors (such as *Lc. rhamnosus* GG or *L. acidophilus* Hambi1448) with supportive *Bacillus* strains offers a novel, synergistic approach to managing PD-related dysbiosis. Consequently, the next phase of this project will focus on proteinase sensitivity assays and the comprehensive characterization and isolation of these cell-free supernatants (CFS) to identify the specific bacteriocins and novel antimicrobial peptides responsible for this targeted inhibition. Future *in vivo* and clinical studies are warranted to validate the efficacy of these specific strains in ameliorating gut dysbiosis and neuroinflammation in PD patients.

## Data Availability

The original contributions presented in the study are included in the article/supplementary material, further inquiries can be directed to the corresponding author.
